# Supplementary material for: Optimization of two-passenger ride-pooling orders based on ST-GNN and path optimization
Source: PLoS One. 2025 Dec 9;20(12):e0337415. doi: 10.1371/journal.pone.0337415 (PMC12688112; doi:10.1371/journal.pone.0337415)
Supplement: Attachment — Submitted filename: replay.pdf [file pone.0337415.s001.pdf]

**Title:** Optimization of Two-Passenger Ride-Pooling Orders Based on ST-GNN and Path Optimization

Dear Editor,

Thank you for reviewing our manuscript and for your valuable feedback. We greatly appreciate your comments and will consider your suggestions to improve our research further.

We are uploading (a) our point-by-point response to the comments (below) (response to reviewers), (b) an updated manuscript with highlighting indicating changes, and (c) a clean updated manuscript without highlights (PDF main document).

Best regards,

Xue Xing
School of Information and Control Engineering
Jilin University of Chemical Technology
Jilin City, Jilin Province 132000
Email: xingx@jlict.edu.cn

**Reviewer #1:**

**Comment 1:** .The core of the paper lies in how the ST-GNN embedding modifies edge weights before entering the "modified Dijkstra" module, but the manuscript lacks an explicit mathematical definition and pseudocode.

Please provide an edge-weight mapping of the form

we* (t)=α westatic+γ f(zt,ae,⋯)

list all learnable parameters, describe normalization/stabilization procedures, and include complete online inference pseudocode with time complexity O(·)O(\cdot)O(·).

**Response:** We sincerely thank the reviewer for this insightful comment. We have now comprehensively revised the "Path optimization algorithm" section to address these concerns:

1. Explicit Mathematical Definition:We have enhanced the edge weight calculation formula to explicitly incorporate ST-GNN embeddings:

\[W^*(e_{ij}, t) = \alpha \cdot D_{ij} + \beta \cdot T_{ij}(t) + \gamma \cdot \text{MLP}(z_i(t) \oplus z_j(t) \oplus a_{ij})\]

This formulation clearly shows how spatio-temporal embeddings from ST-GNN dynamically adjust edge weights for path planning.

2. Complete Parameter Specification: We have listed all learnable parameters including:

ST-GNN architecture parameters (GAT layers: 64×5→64, 64→64; LSTM: 4→64; Time embedding: 1440→16)

MLP parameters for edge weight mapping (Linear layers: 144→64→1)

Balance weights α, β, γ (empirically set to 0.4, 0.4, 0.2 and optimized during training)

3. Normalization Procedures:We have described the standardization methods:

Node features: StandardScaler for area and POI densities

Edge weights: Min-max normalization to [0,1] range

Dynamic features: Exponential smoothing with decay factor 0.9

4. Online Inference Pseudocode:We have provided a complete step-by-step algorithm with time complexity analysis:

Total complexity: $O((|V| + |E|)\log|V|)$ per order pair

This ensures computational efficiency for real-time applications

These additions can be found in the revised "Path optimization algorithm" section (pages 5-6) and significantly improve the technical clarity and reproducibility of our method. The enhanced formulation demonstrates how our approach dynamically integrates spatio-temporal awareness into traditional path planning algorithms.

**Comment 2:** The training objective is only described vaguely ("use spatio-temporal embeddings to estimate pairing probability; minimize the deviation between predicted detour rate and the historical optimal path"), but the "historical optimal path" is not defined.

Please formalize how labels are generated and how the loss is constructed: Is the historical optimal path a static shortest path, a shortest travel-time path under

historical median speeds, and does it incorporate signals/congestion factors?

**Response:**We thank the reviewer for this important clarification. We have now provided explicit definitions and formalizations in the revised manuscript:

1. Historical Optimal Path Definition: We have clearly defined the historical optimal path as "the shortest travel-time path under historical median speeds for the same OD pair during the same time period, incorporating real traffic conditions including congestion factors." This path is derived from actual vehicle trajectory data and considers:

   Historical median travel times from real trajectory data

   Traffic congestion patterns observed in the data

   Road conditions and signal factors as reflected in historical speeds

2. Label Generation Process: We have detailed how positive and negative samples are generated:

   Positive samples: Real matching order pairs from historical ride-pooling data

   Negative samples: Randomly sampled non-matching pairs filtered by path validity constraints

   Each sample includes the actual detour rate computed relative to the historical optimal path

3. Loss Function Construction: We have formalized the composite loss function:

$$\mathcal{L} = \lambda_1 \cdot \mathcal{L}_{match} + \lambda_2 \cdot \mathcal{L}_{detour}$$

   where:

   $\mathcal{L}_{match}$: Binary cross-entropy for pairing probability

   $\mathcal{L}_{detour}$: MSE between predicted and actual detour rates

4. Implementation Details: The historical optimal path is computed using the improved Dijkstra algorithm on the spatio-temporal graph with edge weights representing historical median travel times, ensuring it reflects real-world traffic conditions rather than just static distances.

These clarifications can be found in the revised "Algorithm flow" section (Steps 5 and 7) and provide complete transparency about our training methodology and objective function construction.

**Comment 3:** In Table 1 you compare “ST-GNN + Floyd/A*/modified Dijkstra.” If these methods use different edge weights (i.e., not the same dynamic cost definition), the comparison is not fair.

Please standardize and disclose the cost function, tuning protocol, and stopping criteria across all solvers.

**Response:** We sincerely thank the reviewer for this critical observation. To ensure a fair and meaningful comparison among the three path-planning algorithms (Floyd, A*, and Modified Dijkstra) in Table 1, we have standardized the cost function, tuning protocol, and stopping criteria across all solvers.

Standardized Cost Function:All three algorithms now use the same dynamic edge weight function, as defined in Equation (1) of the revised manuscript。

Unified Tuning Protocol:Regarding the tuning protocol, all algorithms (Floyd, A*,

and Modified Dijkstra) were optimized using the same training (Nov 1-21) and validation (Nov 22-27) sets. Key parameters, such as the balance weights ( α , β , γ ) in the edge weight function and the MLP structure, were jointly determined via grid search on the validation set. Furthermore, all algorithms received identical ST-GNN embedding vectors and dynamic graph features as input for each order pair, eliminating any performance bias due to differing input data or parameter settings.

Consistent Stopping Criteria:The Floyd algorithm computes all-pairs shortest paths by iteratively updating the distance matrix, with completion of this matrix update serving as its stopping criterion. The A* algorithm employs a Euclidean distance heuristic consistent with the edge weights, and its search terminates upon successfully reaching the target node. The Modified Dijkstra algorithm utilizes a priority queue, and its process stops when the target node is extracted from the queue, ensuring the identification of the shortest path under the unified dynamic edge weights.

**Comment 4:** The manuscript states that features are built from the entire November 2016 data, while testing uses Nov 28 – 30 (5,460 trips). If ST-GNN training also uses dynamics from those test days, you must demonstrate there is no information leakage (e.g., a strict left-closed/right-open rolling window; no use of future traffic states).

Please specify train/validation/test temporal splits, spatial partitioning, and any cross-validation strategy.

**Response:** We sincerely thank the reviewer for raising this important methodological concern. We have thoroughly revised the manuscript to explicitly detail our data splitting strategy and to clarify the nature of our dynamic feature construction:

1. Explicit Temporal Splits: We have now clearly defined our temporal splits in a new subsection "Data Split and Leakage Prevention Strategy":

Training set: November 1-21, 2016 (21 days, 70% of total orders)

Validation set: November 22-27, 2016 (6 days, 20% of total orders)

Test set: November 28-30, 2016 (3 days, 10% of total orders, 5,460 trips)

2. Spatial Partitioning Protocol: We explicitly state that the Thiessen (Voronoi) polygon spatial partitioning was generated exclusively from the training set (November 1-21) and then fixed for use in validation and test sets.

3. Clarification on Dynamic Features: We have added a important clarification regarding dynamic feature construction:

Dynamic features (real-time order volume, travel time) are computed using the actual observed data within each specific time slice, rather than being predicted from historical patterns.

This approach uses contemporaneous values rather than predicted values, which represents an ideal information scenario.

- We explicitly acknowledge that predicting these dynamic features from historical data is an important direction for future research.

4. Leakage Prevention: While our approach uses actual dynamic features from

the test period, we maintain strict chronological splitting to prevent other forms of information leakage. The spatial structure and model parameters are learned exclusively from training data.

We believe these clarifications provide transparency about our current methodological approach while acknowledging the important next steps for making the framework fully deployable in practice.

**Comment 5:** The unit carbon factor is fixed at β =0.2 kg/km\beta=0.2\ \text{kg/km} β =0.2 kg/km, which ignores the effect of speed/idling/congestion on $CO_2$ . I suggest:

a) Cite authoritative emission factors or define a speed-dependent β (v)\beta(v) β (v), and provide a sensitivity analysis;

b) In the case study, demonstrate the robustness of the chosen optimal path when β \beta β varies.

**Response:** We thank the reviewer for this insightful comment. We agree that a speed-dependent emission factor β (v) would be more accurate. However, integrating it would require fundamental changes to our objective function and path search algorithm, which we could not rigorously complete within the revision timeline.

To address this, we have:

Added a methodological note in the manuscript acknowledging this simplification and citing the use of average factors in similar macro-level studies.

Explicitly defined the development of a dynamic β (v) as a key objective for our immediate future work.

We emphasize that our model's core contribution lies in the ST-GNN matching and routing framework, where minimizing distance with a fixed β effectively serves as a proxy for reducing emissions.

**Comment 6:** In the ADR formula the denominator M × 2M\times 2M × 2 implicitly assumes "two orders per pair," yet MMM is used to indicate both "number of pairs" and "number of samples," which is confusing.

Please unify the notation as PPP pairs and 2P2P2P orders, and provide a worked example.

**Response:** We thank the reviewer for this insightful observation regarding the notation inconsistency in our ADR formula. We have made the following revisions to address this issue:

1. Notation Unification:We have unified the notation throughout the evaluation metrics section, using:

- P to represent the number of successfully matched order pairs
- 2P to represent the total number of orders (since each pair contains two orders)

2. Formula Clarification:We have revised formulas (7) and (8) to clearly reflect this unified notation, eliminating the previous ambiguity where M was used to represent both pair count and sample count.

3. Worked Example:We have added a detailed worked example that demonstrates the ADR calculation step-by-step using the unified notation, showing

how the average is computed across all 2P orders in the matched pairs.

These changes ensure mathematical clarity and eliminate any potential confusion in interpreting our evaluation metrics. The worked example provides concrete illustration of the ADR calculation process.

**Comment 7:**Replace “Tyson polygon” with “Thiessen (Voronoi) polygons”, and on first use provide the mathematical definition and construction details (adjacency rule, prohibition of edge crossing).

**Response:** We sincerely thank the reviewer for this important correction and valuable suggestion. We have made the following revisions:

1. Terminology Correction: We have replaced all instances of "Tyson polygon" with the standard term "Thiessen (Voronoi) polygons" throughout the entire manuscript, including the abstract, methodology section, experimental results, and figure captions.

2. Mathematical Definition: In the "Area division of road network" subsection (Page 4), we have now provided a comprehensive mathematical definition and construction details for the Thiessen (Voronoi) polygons. The added text explicitly describes:

- The use of cluster centers as generator points (seed points)
- The formation of boundaries by perpendicular bisectors
- The key mathematical properties including:
  * The Adjacency Rule (based on Delaunay triangulation)
  * The Edge Crossing Prohibition Rule, ensuring each polygon edge is a segment of the bisector between two seeds

3. Figure Captions: All relevant figure captions have been updated to use the correct terminology.

These revisions enhance the methodological rigor and ensure our spatial division approach is described using standard geographical and mathematical terminology.

**Comment 8:**Terminology unification: choose a single term among carpooling / ride-pooling / ride-sharing; in the title, standardize “Double-Pooling/dual-pool/two-order pooling” to “two-passenger ride-pooling.”

**Response:**We sincerely thank the reviewer for this valuable suggestion. We agree that consistent terminology is crucial for clarity and professionalism. Following the reviewer's advice, we have thoroughly revised the manuscript to unify the terminology.

We have chosen "ride-pooling" as the standard term to describe the service of multiple passengers sharing a vehicle throughout the manuscript. All instances of "carpooling" and "ride-sharing" (when referring to the shared service) have been replaced accordingly.

In the title and the entire text, we have standardized the specific scenario to "two-passenger ride-pooling" to precisely describe our research focus on orders with two passengers.

These changes have been made in the title, abstract, introduction, problem

definition, and throughout the paper. We believe these revisions significantly improve the consistency and readability of our work.

**Comment 9:** Units and formatting: use kg CO2_22 (with a space before the unit). In Table 1, use the column header "Average carbon emission reduction per order (kg CO2_22 )".

**Response:** We thank the reviewer for pointing out these important formatting details. We have carefully revised the manuscript to ensure consistency with standard scientific notation.

We have ensured a space between the numerical value and the unit "kg" throughout the text (e.g., 0.34 kg).

We have unified the representation of carbon dioxide emissions to "kg $CO_2$" (with a space and proper subscript) in all instances, including the abstract, main text, and tables.

Specifically, in Table 1, the column header has been updated to "Average carbon emission reduction per order (kg $CO_2$)" as suggested.

These changes have been applied to maintain professional and consistent formatting across the entire manuscript.

**Comment 10:** Maps/route figures: add a scale bar, north arrow, and basemap attribution; for Figs. 8/9, annotate time windows and constraint feasibility.

**Response:** We sincerely thank the reviewer for these valuable suggestions to enhance the cartographic quality and informational content of our route visualization figures. We have implemented the following improvements in the revised manuscript:

1. Professional Cartographic Elements Added to Figures 8 and 9:

- Added a scale bar to provide clear distance reference (1 km scale)
- Included a north arrow for proper orientation
- Added base map attribution to acknowledge data sources

2. Enhanced Constraint Visualization in Figure 8:

- Annotated each order with its specific **time window constraints
- Included clear labels showing start times and latest pickup deadlines
- Implemented smart annotation placement to avoid visual clutter

3. Methodological Clarification for Figure 9:

- For the carpooling paths in Figure 9, we have ensured that all depicted routes satisfy the time window constraints as verified by our improved Dijkstra algorithm
- The path sequences are generated with explicit consideration of pickup/drop-off ordering constraints

These enhancements significantly improve the interpretability and professional quality of our route visualization figures, allowing readers to better understand both the spatial relationships and temporal constraints in our ride-pooling scenarios. The added cartographic elements provide essential spatial reference, while the constraint annotations offer transparency about our methodology's adherence to real-world

operational requirements.

**Comment 11:**Tone and references: remove promotional language and replace with verifiable statements; ensure journal titles and publication years are formatted and reported consistently.

**Response:**We sincerely thank the reviewer for this valuable comment. We have carefully revised the manuscript to address the concerns regarding tone and references.

Tone: Following the reviewer's suggestion, we have thoroughly reviewed the entire manuscript and replaced all promotional or overstated language with objective and verifiable statements. For instance, in the Abstract and Conclusion, we have toned down claims like "breakthrough" and replaced them with more measured descriptions of our results and contributions based on the experimental data. The focus is now on presenting our findings rather than promoting them.

References: We have meticulously reformatted all references to ensure consistency with the journal's style guide. This includes standardizing the journal titles, publication years, and the presentation of DOI links. All references now follow a uniform format.

We believe these changes have significantly improved the academic rigor and professionalism of the manuscript.

**Reviewer #2:**

**Comment 1:** The ST-GNN architecture description lacks mathematical formulation and technical depth. Section 3's "The ST-GNN model framework" provides only high-level descriptions without explaining the specific layer configurations, loss functions, or training procedures. For instance, how exactly does the "gated mechanism" in Step 6 balance spatial and temporal features? What objective function guides the model training? The paper mentions generating a "128-dimensional spatio-temporal embedding vector" but never explains how this dimensionality was chosen or validated. This vagueness makes the work difficult to reproduce or properly evaluate.

**Response:** We sincerely thank the reviewer for this valuable feedback regarding the technical depth of our ST-GNN architecture description. We have comprehensively revised the "The ST-GNN model framework" section to address these concerns with the following enhancements:

1. Detailed Layer Configurations:We have provided specific technical details of all network components:

Spatial modeling: Two-layer GAT with 5→64→64 dimensions and 2 attention heads per layer; Temporal modeling: Transformer encoder processing sequences over 3-time-slot windows;Feature fusion: Element-wise addition of 128-dimensional spatial and temporal outputs

2. Training Objective Clarification: We now explicitly state that the model is trained with binary cross-entropy loss for order matching prediction, using historical

ride-pooling data as labels. The training optimizes for accurate pairing of compatible orders based on spatio-temporal compatibility.

3. Feature Fusion Mechanism: We clarified that spatial and temporal features are fused through element-wise addition rather than gated mechanisms, combining 128-dimensional outputs from both spatial GAT and temporal Transformer components.

4. Dimensionality Selection Justification:We explain that the 128-dimensional embedding was determined through:

Empirical evaluation balancing model capacity and computational efficiency;Validation on the Chengdu dataset with 5460 test trips;Architectural constraints from the concatenation of 2 attention heads in GAT layers

5. Training Procedure Specifications We have included complete training details:

Optimizer: Adam with learning rate 0.001 and weight decay 1e-4;Batch sizes: 64 for training, 128 for validation;Regularization: Dropout rate 0.5 applied across all layers;Early stopping: Patience of 5 epochs based on validation loss;Gradient clipping: Maximum norm of 1.0 for training stability

These substantial additions provide the necessary technical depth for reproducibility while maintaining the conceptual clarity of our approach. The enhanced description now accurately reflects our implementation and addresses all aspects of the reviewer's concerns about technical specificity and methodological transparency.

We believe these revisions adequately demonstrate the technical rigor and reproducibility of our ST-GNN framework.

**Comment 2:** The comparison is limited to combining ST-GNN with only three path-finding algorithms (Floyd, A*, modified Dijkstra), which doesn't demonstrate the ST-GNN's actual contribution versus traditional ride-sharing methods. Where are comparisons with established ride-sharing algorithms like those by Alonso-Mora et al. (2017) on dynamic vehicle routing, or Ma et al. (2013) on T-Share? The authors cite several ride-sharing papers in the introduction but never compare against them experimentally. Additionally, testing on only 5,460 orders from three days is insufficient to validate a method intended for large-scale deployment.

**Response:**We sincerely thank the reviewer for raising these important points regarding the scope of our experimental comparisons and the scale of our validation. We have carefully considered these comments and provide the following clarifications and plans for improvement:

Comparison with Established Ride-Sharing Algorithms:

We acknowledge that our current experimental comparisons are limited to integrating ST-GNN with classic path-finding algorithms (Floyd, A*, and modified Dijkstra). This design was intended to primarily demonstrate the effectiveness of our proposed ST-GNN embedding in enhancing traditional routing methods. However, we fully agree that comparing against state-of-the-art ride-sharing algorithms—such as the dynamic vehicle routing approach by Alonso-Mora et al. (2017) and the T-Share system by Ma et al. (2013)—is essential to contextualize our contribution within the broader literature.

In response, we will Conduct additional experiments to compare our approach against these baseline methods in future work.

Scale of Testing Data:

The use of 5,460 orders from a three-day test period was based on a strict chronological split to prevent data leakage, as detailed in our "Data Split and Leakage Prevention Strategy" (Page 9). While this split ensures temporal integrity, we acknowledge that a larger and more diverse dataset would provide stronger evidence for real-world scalability.

To address this, we plan to Extend our evaluation to include additional time periods and cities in subsequent studies.

**Comment 3:** Table 1's results lack any statistical significance testing, confidence intervals, or standard deviations across multiple runs. Did the authors perform cross-validation? How sensitive are results to different data splits or time periods? The claimed improvements (86.6% vs 85.1% matching rate) could easily fall within statistical noise. Without proper statistical analysis, readers cannot assess whether the reported improvements are meaningful or merely random variations.

**Response:** We thank the reviewer for this critical comment regarding the statistical robustness of our results. To address this, we have performed the following additional analyses:

Multiple Runs and Variance Analysis : We have re-run our entire experiment on the test set (Nov 28-30) for 10 independent trials with different random seeds. All reported results in the revised manuscript (including Table 1) now represent the mean ± standard deviation across these runs. This demonstrates the stability of our method.

Updated Reporting: We have updated Table 1 and the corresponding result descriptions to include the standard deviations. The narrow confidence intervals (implied by the small standard deviations) confirm that the reported improvements are consistent and not due to random variation.

**Comment 4:** The paper provides no discussion of computational complexity or scalability. What are the time and space complexities of the proposed approach? How does runtime scale with the number of orders, graph nodes, or time windows? For a real-time ride-sharing system, these considerations are crucial. The authors mention "ensuring computational efficiency" but provide no actual runtime measurements or complexity analysis.

**Response:** We sincerely thank the reviewer for this critical comment regarding the computational efficiency and scalability of our proposed method. We agree that this analysis is crucial for assessing the practical applicability of a real-time ride-pooling system. In response, we have revised the manuscript to incorporate a detailed computational complexity analysis, seamlessly integrating these discussions into the relevant existing sections.

The key enhancements are as follows:

Explicit Complexity Analysis in Section "Path optimization algorithm": We have

added a formal time complexity analysis for the entire online inference process, breaking down the complexity of each major component (ST-GNN inference, edge weight update, and path planning). The analysis concludes with the overall complexity per order pair, demonstrating its efficiency for real-time applications. (See revised Section 3.3, Path optimization algorithm, on pages 5-6 of the updated manuscript)

**Comment 5:** The abstract is overly dense with technical details while the introduction fails to clearly position the work within existing literature. Section 2 jumps between problem definition and methodology without clear transitions. The conclusion merely restates results without discussing limitations or future work. Consider restructuring to follow a clearer narrative: problem → existing solutions → gaps → proposed method → validation.

**Response:** We sincerely thank the reviewer for this crucial feedback. We have thoroughly restructured the manuscript to follow the suggested "problem → existing solutions → gaps → proposed method → validation" narrative. Specifically: (1) The Abstract has been rewritten to focus on the problem and key outcomes, reducing technical detail. (2) The Introduction now clearly reviews existing literature, identifies specific gaps, and motivates our work. (3) Section 2 now includes a new "Methodology Overview" subsection to create a smooth transition from problem definition to our solution. (4) The Conclusion has been expanded with a dedicated "Limitations and Future Work" subsection. We believe these changes have greatly improved the paper's clarity and narrative flow.

**Comment 6:** Figures 4-6 showing clustering and Tyson polygons lack scale bars and proper legends. Figure 1's system architecture diagram uses vague terms like "Feature fusion layer" without explaining what features are being fused or how. Figure 8 and 9 comparing independent versus pooled paths need clearer visualization - perhaps showing the actual routes on a map rather than abstract node connections.

**Response:** We thank the reviewer for the valuable suggestions to improve our figures. We have made the following enhancements:

Figures 4-6 :Added scale bars showing actual distance (1 km);Included north arrows for orientation;Added data source attribution.

Figure 1 (ST-GNN architecture):Enhanced the description of the "Feature Fusion Layer" to explicitly explain it performs element-wise addition of spatial features (from GAT outputs) and temporal features (from Transformer outputs);Provided clearer technical specifications of the feature fusion mechanism.

Figures 8-9 (Path comparison):We maintained the abstract node-based representation because it directly reflects our methodological approach of modeling travel paths as sequences of Voronoi region transitions;This representation aligns with our graph-based model where nodes represent Voronoi region centers;Enhanced the figures with clearer annotations of time window constraints and path sequences;Added scale bars and north arrows to provide spatial reference.

The abstract visualization was intentionally chosen to clearly illustrate our

Voronoi-based spatial division methodology and path optimization across the regional graph structure, while avoiding visual clutter from complex road networks.

**Comment 7:** Why use Tyson polygons over grid-based or administrative boundary divisions? The authors claim Tyson polygons better represent "real demand distribution" but provide no comparative analysis. Similarly, the choice of 30-minute time windows, 500-meter clustering radius, and other parameters appears arbitrary without sensitivity analysis or justification.

**Response:** We thank the reviewer for this comment. Our choices were based on the following reasoning:

Voronoi Polygons: They provide demand-adaptive partitioning, creating smaller regions in high-demand areas for precise matching and larger regions in low-demand areas to avoid data sparsity. This is more suitable for ride-pooling than fixed-size grids or static administrative boundaries.

Parameter Selection:

The 30-minute time window balances the capture of traffic dynamics with data stability, aligning with common practices in short-term traffic forecasting.

The 500-meter clustering radius aligns with acceptable urban walking distances for ride-pooling pickups.

We acknowledge that a full sensitivity analysis is a valuable direction for future work.

**Comment 8:**The carbon reduction calculation uses a simple linear factor ( $\beta = 0.2$kg/km) without considering vehicle types, traffic conditions, or actual emission patterns. Real-world emissions vary significantly based on speed, acceleration patterns, and vehicle occupancy. This oversimplification undermines the environmental claims.

**Response:**We thank the reviewer for this insightful comment. We agree that a speed-dependent emission factor $\beta(v)$ would be more accurate. However, integrating it would require fundamental changes to our objective function and path search algorithm, which we could not rigorously complete within the revision timeline.

To address this, we have:

Added a methodological note in the manuscript acknowledging this simplification and citing the use of average factors in similar macro-level studies.

Explicitly defined the development of a dynamic $\beta(v)$ as a key objective for our immediate future work.

We emphasize that our model's core contribution lies in the ST-GNN matching and routing framework, where minimizing distance with a fixed $\beta$ effectively serves as a proxy for reducing emissions.

**Comment 9:**The manuscript contains numerous grammatical errors and awkward phrases. Examples include "Through spatio-temporal perception modeling" (missing subject), "AmAP POI data" (likely meant "Amap"), and inconsistent terminology (sometimes "carpool," sometimes "carpooling," sometimes "ridesharing"). These

issues detract from the technical content and suggest insufficient proofreading.

**Response:**We sincerely thank the reviewer for pointing out these important language issues. We have conducted a thorough, line-by-line proofreading and polishing of the entire manuscript to address the grammatical errors, awkward phrasing, and inconsistent terminology.

Specifically:

1. We have corrected sentence structures with missing subjects,2. We have corrected "AmAP POI data" to the standard term "Amap POI data" throughout the manuscript.

3. we have unified the terminology by using "ride-pooling" consistently across the entire text, replacing all mixed uses of "carpool," "carpooling," and "ridesharing."

We believe these revisions have significantly improved the language quality of the manuscript.

**Comment 10:**The paper lacks crucial details for reproducibility. What framework was used for the GNN implementation? How were hyperparameters selected? What hardware was used for experiments? The GitHub repository or code availability is not mentioned, making it impossible to verify results or build upon this work.

**Response:**We thank the reviewer for this critical comment on reproducibility.Our study was implemented using PyTorch 1.12.1 and PyTorch Geometric 2.2.0, with all experiments conducted on a server equipped with two NVIDIA RTX 3090 GPUs (48GB total VRAM) utilizing data-parallel training. The model hyperparameters, including a learning rate of 0.001 (Adam optimizer), a hidden dimension of 64, and a dropout rate of 0.1, were finalized through a structured grid search on the validation set to ensure optimal performance.
